# ORCID

**DOI:** 10.5195/jmla.2017.89

**Published:** 2017-04

**Authors:** Evan R. Sprague

## PURPOSE

ORCID is a not-for-profit organization that provides the Open Researcher and Contributor ID (ORCID), allowing researchers to register for a unique identifier. ORCID’s mission describes the service as providing “an identifier for individuals to use with their name as they engage in research, scholarship, and innovation activities” [1]. These identifiers are primarily used to disambiguate authors in the publishing world.

## DESCRIPTION

Launched in 2012, ORCID provides unique alphanumeric identifiers to researchers with the goal of providing authority control for authors in the scientific and academic communities. With an ORCID identifier, authors can distinguish themselves from others with similar or identical names. For example, two John M. Smiths can be distinguished by assigning separate ORCID identifiers, making it much easier to locate works by either of them. ORCID also allows information systems to better identify authors and link them to their works.

ORCID primarily promotes itself to researchers and institutions. ORCID allows researchers to take advantage of its unique identifiers and allows institutions to assist their members with profile upkeep. Recently, some publishers have begun to require or request that authors include an ORCID identifier when publishing in their journals to make it easier for the publisher to disambiguate authors from one another. ORCID identifiers are also being indexed in major citation databases such as Scopus, Web of Science, and PubMed.

## SEARCHING ORCID

ORCID’s website has a very simple layout. A search bar at the top of the site allows users to search for researchers with existing ORCID identifiers. Selecting the gear icon next to the search bar will navigate users to an advanced search page. From the advanced search page, users can search by ORCID identifier, first name, last name, and keyword. With a keyword search, users can find researchers who work in a particular field or have works in a specific topic area. Such a search could be especially useful for someone looking for collaborators. A researcher’s profile page shows the researcher’s education, employment history, and authored works.

## ORCID AND RESEARCHERS

ORCID identifiers are free for researchers, and signing up is quick and easy, simply requiring users to fill out a short registration form. An ORCID identifier is associated with the researcher, not a place of employment or a particular field. As a result, ORCID identifiers can follow researchers throughout their careers. Having all works listed in one location can also be advantageous, allowing others to see researchers’ works across multiple fields and in a variety of formats. By linking to their ORCID profiles, such as on a CV or personal website, researchers can easily share their complete lists of works. Because the identifier is unique to an individual, it also allows works to be tracked before and after a name change, a big advantage to those who change their names during their careers.

One of the major advantages of ORCID identifiers is the wide array of works that researchers can add to their profiles, including articles, newsletters, conference posters, lectures, videos, datasets, and many more. Each of these can be entered easily by researchers through a simple template provided in the profile. In addition to these works, researchers can also record and display grants and other funds that they have been awarded. Researchers can alter the visibility settings for each item listed in their profiles to either public, trusted parties (those to whom one has given permission), or private (only visible to the researcher). These settings are located to the right of each citation or information piece.

## ORCID AND ORGANIZATIONS

Through a paid membership fee, an organization can become an ORCID member. The fee for a basic membership is $5,000 for a standard single legal entity. These organizational memberships have 2 major benefits. The first is access to ORCID application programming interfaces (APIs), which can help an organization create ORCID identifiers for the members of the institution, for example, by creating emails that include links to the ORCID registration form with some of the fields prepopulated for users. This makes an already short process even easier. It also prompts users to allow the organization to access their accounts to add items, the second benefit of becoming a paid ORCID member. As long as researchers have granted access, specified individuals in the organization can add works to the researchers’ accounts. This privilege can be revoked by researchers at any time. The ability for an organization to edit profiles can be very useful when first implementing ORCID identifiers among an entire department or organization. By taking advantage of ORCID’s citation listings, an organization will be better able to track and maintain their researchers’ works.

## SIMILAR PRODUCTS

The only other large authority control product for researchers that is still active is the Thomson Reuters ResearcherID, which also offers users a unique identifier for the purpose of disambiguation. A ResearcherID can be linked to an ORCID identifier and vice-versa. Over the past few years, there have been other attempts to provide author authority control, for example, ScopusID and the PubMed Author ID Project. However, most of these projects, including both ScopusID and the PubMed Author ID Project, have been discontinued, and they now instead urge their users to register for an ORCID identifier.

## ISSUES WITH ORCID

The biggest issue with using an ORCID identifier is simply keeping it up-to-date. If an organization is willing to invest in a membership and provide support, upkeep may be less of an issue for researchers. Also, unlike institutional citation tracking systems, researchers may be more invested in keeping their ORCID profiles updated since the list of works can follow them through their careers. The other issue is the likelihood of ORCID becoming a lasting figure in the field of author authority control. As so many other attempts have failed, ORCID has emerged as a leader in the field, making it reasonable to assume that ORCID will be around a while.

## CONCLUSION

Differentiating between authors with similar names or tracking researchers who have changed their names has been an ongoing issue in the scholarly and publishing realm. ORCID identifiers provide researchers, publishers, and organizations a simple and easy way to track an individual’s complete scholarly history. With publishers requesting ORCID identifiers and citation databases indexing ORCID identifiers, having an ORCID identifier is becoming more appealing to those wishing to publish. Regardless of whether an organization invests in a membership, obtaining an ORCID identifier is well worth an individual researcher’s time and effort to sign up and maintain a free account.
